# Establishment of a long-term secondary lymphedema animal model in the rodent hindlimb

**DOI:** 10.3389/fsurg.2025.1703868

**Published:** 2026-01-16

**Authors:** Sung-Hwan Yoon, Hyun Suk Peter Suh, Jin-Hui Yoon, Hayeong Cho, Yujin Myung, Jae Yong Jeon

**Affiliations:** 1 Department of Plastic and Reconstructive Surgery, Seoul National University Bundang Hospital, Seongnam, Republic of Korea; 2 Department of Plastic and Reconstructive Surgery, Asan Medical Center, University of Ulsan College of Medicine, Seoul, Republic of Korea; 3 Center for Vascular Research, Institute for Basic Science, Daejeon, Republic of Korea; 4 Department of Rehabilitation Medicine, Asan Medical Center, University of Ulsan College of Medicine, Seoul, Republic of Korea

**Keywords:** secondary lymphedema, lymphatic system, lymphangiogenesis, translational model, lymphangiography

## Abstract

**Introduction:**

Secondary lymphedema is a chronic swelling of the extremities caused by physical disruption of the lymphatic system, leading to impaired lymph drainage. It frequently develops in cancer patients after surgical removal of lymph nodes and vessels during tumor resection, when reconnection of lymphatic pathways fails. Current clinical therapies are mainly palliative or conservative, with limited therapeutic effects. Therefore, an animal model that closely mimics the chronic and severe characteristics of secondary lymphedema in patients is required to enable mechanistic and therapeutic research.

**Methods:**

To establish a long-term secondary lymphedema model in the mouse hindlimb, a combination of surgical, radiological, and mechanical interventions was designed. A novel surgical procedure termed the folding suture technique was developed to disrupt both the superficial and deep lymphatic networks. Controlled radiation exposure was applied postoperatively to inhibit early-stage lymphangiogenesis, while hindlimb immobilization was introduced to suppress lymphatic pumping and enhance edema formation.

**Results:**

The newly developed model showed a significant and persistent increase in hindlimb paw thickness, with edema sustained for over six weeks. Immunofluorescence analysis demonstrated a markedly reduced number and diameter of regenerated lymphatic vessels compared to previously established models. Functional lymphography using fluorescein isothiocyanate (FITC)-dextran and live indocyanine green (ICG) imaging confirmed diminished lymphangiogenesis and impaired lymphatic flow. Further evaluation using the leg dermal backflow (LDB) staging system—commonly applied in clinical assessment—showed consistently higher severity scores, indicating a robust and irreversible secondary lymphedema phenotype.

**Discussion:**

This study demonstrates that the newly established mouse hindlimb lymphedema model successfully replicates the chronic, severe, and irreversible nature of clinical secondary lymphedema. The combination of the folding suture technique, radiation-induced inhibition of lymphangiogenesis, and immobilization effectively induces and maintains the pathology. This model provides a reliable preclinical platform for in-depth investigation of secondary lymphedema pathophysiology and for the development and validation of novel therapeutic strategies.

## 1 Introduction

Lymphedema is swelling of the upper or lower extremities caused by accumulation of excessive fluids in the interstitial flow. In particular, secondary lymphedema primarily occurs due to structural damage of the lymphatic system, such as surgical removal of lymph nodes and lymphatic vessels as part of the treatment procedure for cancer to prevent metastasis. Lynch et al. (1) reported that lymph node dissection in cancer treatment is responsible for secondary lymphedema and the severity of edema significantly increases after radiation therapy. Moreover, previous studies (2, 3) have reported that upper and lower extremity lymphedema cases occur in 16% to 39% of breast cancer patients and 20%–49% of genital cancer patients. There are a number of clinically available surgical and non-surgical therapeutic strategies to treat secondary lymphedema. Lymphovenous anastomosis and vascularized lymph node transfer are commonly practiced as surgical treatments, and complete congestive therapy is widely prescribed as a non-surgical treatment for lymphedema (4, 5). However, due to the complexity of the lymphatic system and its vasculature, both surgical and non-surgical treatments have limited clinical outcomes. Therefore, lymphedema treatment is generally regarded to be a palliative, providing only temporary relief of symptoms and preventing its progression (6).

To understand the pathophysiology of the lymphatic system and to develop effective treatments for secondary lymphedema, it is essential to establish a standardized long-term secondary lymphedema animal model that has high correlation to human patients. A number of previous studies have reported the development of lymphedema hindlimb models by surgically removing popliteal and/or inguinal lymph nodes (7–9) or by applying controlled radiation (6, 10). However, these animal models exhibit major limitations in both the severity and duration of lymphedema, as they produce only short-term manifestations of secondary lymphedema. This restriction has hindered further studies into the pathophysiology of secondary lymphedema and the development of therapeutic strategies.

We hypothesized that complete disruption of both collecting and superficial lymph flow is essential for developing an effective long-term lymphedema animal model. To create an animal model highly correlated with lymphedema patients, a combination of a newly developed surgical technique, controlled radiation exposure, and immobilization technique was applied. Thus, the purpose of this study was to create a long-term secondary lymphedema animal model in the rodent hindlimb. In order to evaluate the successful establishment of secondary lymphedema on a hindlimb, we performed histological analysis, including measurement of hindlimb paw diameter, dermis and subcutaneous layer thickness, and quantification of lymphatic vessels and capillaries. Immunofluorescence (IF) confocal microscopy was employed to selectively visualize and analyze regenerated lymphatic vessels to evaluate lymphangiogenesis and analyze the condition of the regenerated lymphatic vessels after the procedure. For a thorough, in-depth evaluation of dermal backflow and condition reconnection of the lymphatic system and its flow, both lymphography and ICG lymphangiography were performed, which validated that the original cause of edema was solely attributable to the disruption of the lymphatic system and its drainage dysfunction.

## 2 Materials and methods

All animal study procedures were thoroughly reviewed and approved by the Institutional Animal Care and Use Committee (IACUC) of the Asan Institute for Life Sciences (Asan Medical Center, Seoul, Korea) and the IACUC of KAIST (KAIST, Daejeon, Korea). All experiments were conducted in strict accordance with the US National Institute of Health guidelines for the care and use of laboratory animals.

### 2.1 Rodent lymphedema hindlimb model

Ten-week-old male C57BL/6 (Orient Bio, Seong-nam, Republic of Korea) mice were used to create the lymphedema hindlimb model, as described in previous studies (7, 11, 12). The procedure was performed on both control and the experimental groups using different surgical techniques.

**Figure 1:**
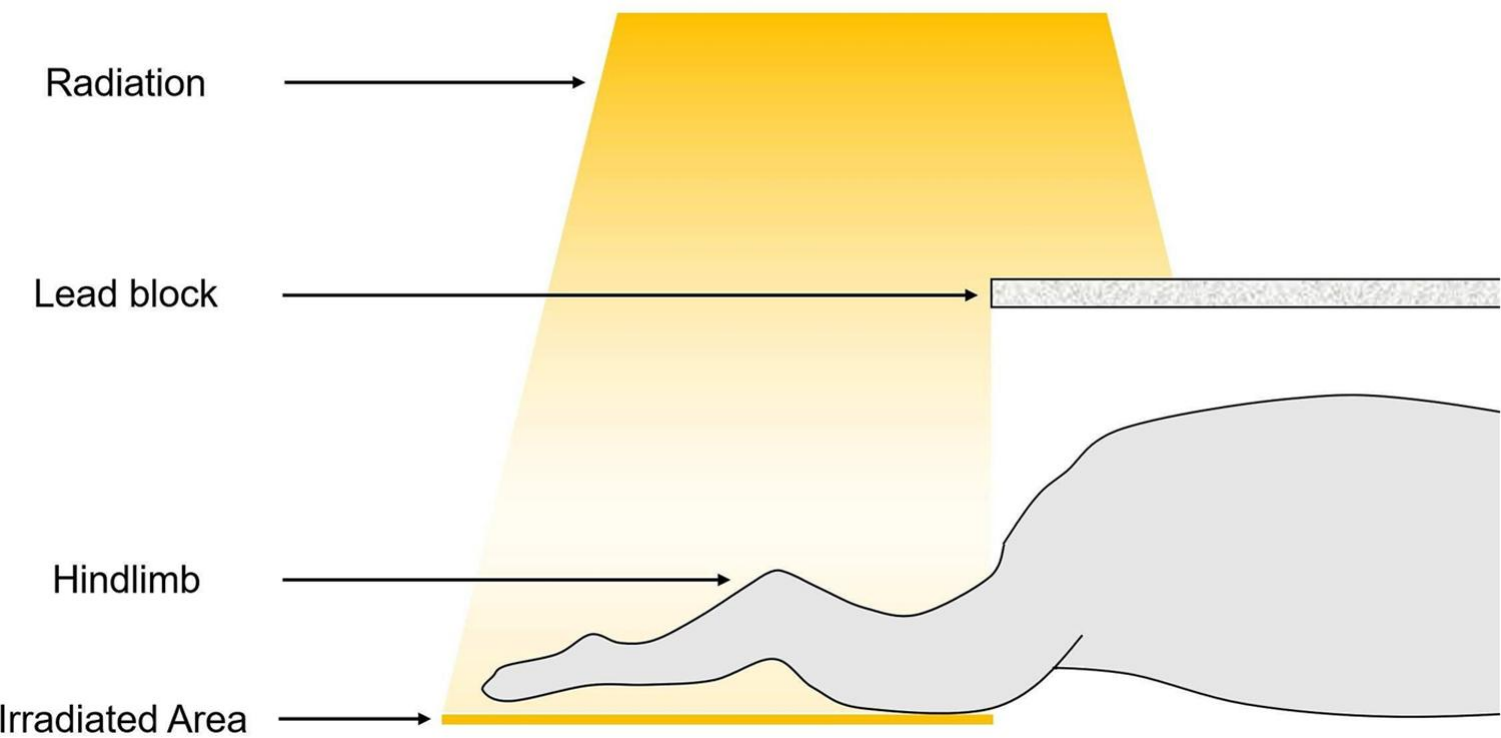
Schematic illustration of radiation procedure when applying controlled radiation when creating a preclinical lymphedema model. To avoid unnecessary radiation overexposure to accomplish successful establishment of lymphedema model and warranting survival of the animals, lead blocks were applied to cover proximal to the lower extremities. Note that a total of 10 Gy radiation was applied at a dose rate of 4.12 Gy/min for optimal results.

For the surgical procedure, all mice were anesthetized using an intramuscular injection of 50 mg/kg zolazepam and tiletamine (Zoletil 50; Virbac, Carros, France) and 10 mg/kg xylazine (Rompun; Bayer HealthCare, Leverkusen, Germany). Then, hair on both sides of the hindlimb was completely removed using ER-1511 electric shaver (Panasonic, Osaka, Japan) and human-grade shaving cream (Nair, Florida, USA) for clear visualization of the lower extremities for accurate edema assessment. The skin was washed and sanitized with Povidone-iodine solution for disinfection, and a circumferential skin incision of the dermis was performed 1 cm from the knee joint without damaging major blood vessels. To visualize the lymphatic system, 0.1 mL of 1% Evans blue in PBS was injected intradermally. Excision of the popliteal lymph node was performed, followed by cauterization of both proximal and distal ends of the popliteal lymph node using Bovie cautery. After excision, the incision site was sutured with 7-0 silk sutures. The mice in the control group were sutured using a conventional simple interrupted suture. The mice in the experimental group were sutured using a newly developed inverted interrupted suture pattern called a “folding suture” method, described in detail in the following. One day after the procedure, the lower limb region in both the control and experimental groups was exposed with 10 Gy, as proposed by previous studies, to a single dose of controlled radiation using the X-RAD 320 (Precision X-Rad, Connecticut, USA) at a dose rate of 4.12 Gy/min (150 kVp, 20 mA) (13, 14). The rest of the body was completely shielded from radiation using lead blocks to avoid unnecessary radiation overexposure (Figure 1). All mice received necessary post-surgical treatment, were housed in a temperature-controlled room (22 °C ± 2 °C), and provided with food and water *ad libitum*.

A total of 36 mice were assigned to each group for this study to ensure adequate power calculation and statistical significance. In each group, 18 mice were used for hindlimb paw thickness measurement and histological analysis (H&E staining and immunofluorescence staining) and the other 18 mice were used for ICG lymphography and real-time lymph flow analysis. To ensure accuracy of the data collection, hindlimb paw thickness measurements were performed prior to histological analysis.

### 2.2 Folding suture and immobilization

**Figure 2:**
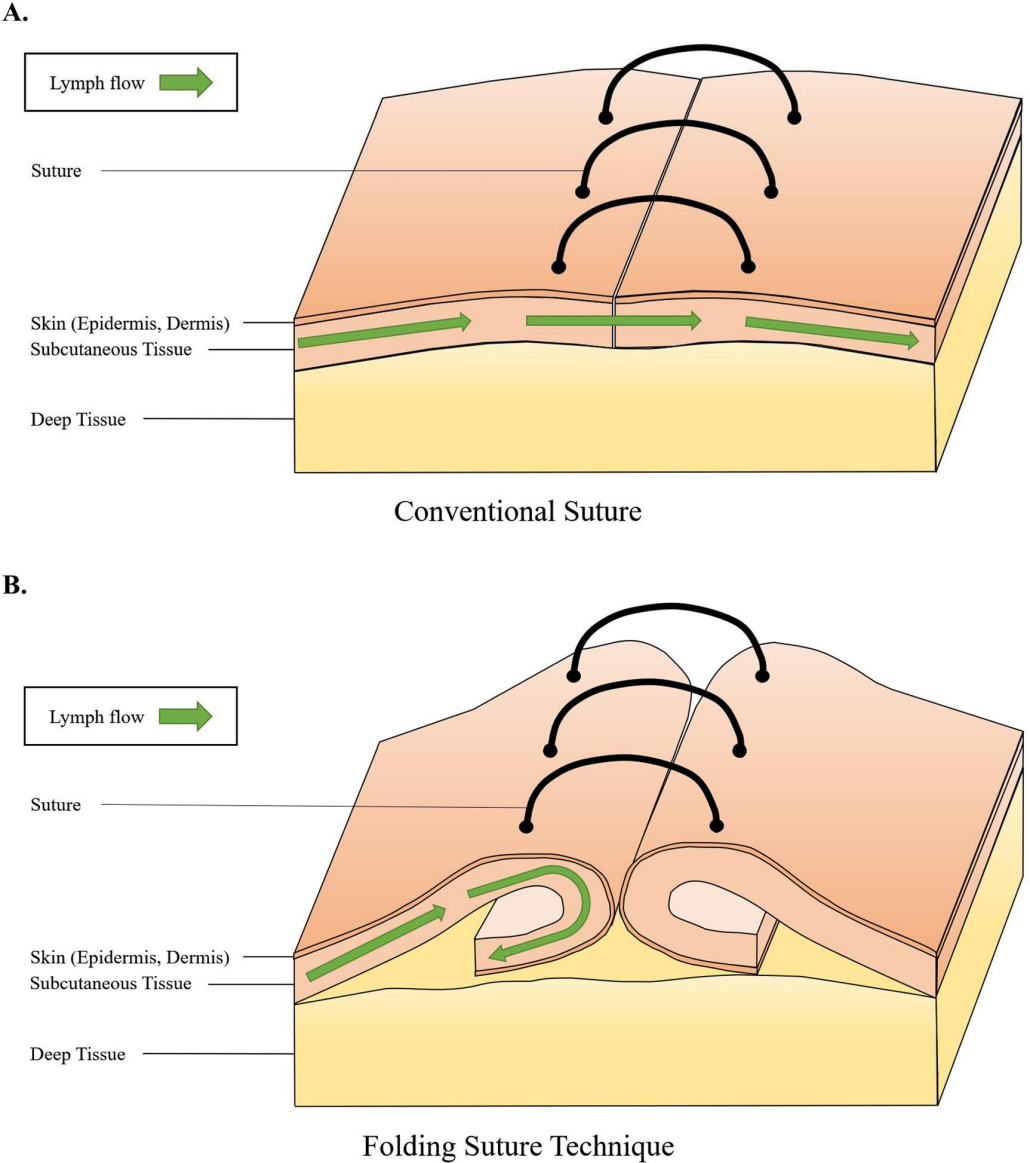
**(A)** Schematic illustration of a conventional suture used in previously established lymphedema hindlimb models. The superficial lymphatic system is located in the epidermis, dermis, and subcutaneous tissue region and it is responsible for lymph flow. When using the conventional suture, the superficial lymphatic system and its lymphatic flow recover instantly when the healing process of these regions is complete, and accumulation of lymph flow and progression of lymphedema are successfully completed. **(B)** Schematic illustration of a newly designed and established folding suture technique. When using this surgical technique, the surgically incised surface of skin and subcutaneous tissue is not intact, causing a delay in recovery process of the superficial lymphatic system and its lymphatic flow. In the meantime, delayed recovery time allows induction of accumulation of lymph flow and progression of lymphedema; this eventually triggers irreversible tissue changes and causes chronic lymphedema even after the healing process is complete.

The folding suture is a newly developed suturing technique based on a conventional inverted interrupted suture method that effectively disrupts the interstitial flow of the superficial lymphatic system. This method successfully blocks lymphatic drainage in an early stage before the wound healing process is complete, resulting in significantly decreased of the lymph flow capacity and causing long-term chronic secondary lymphedema in hindlimbs due to acute edema. This method is based on the inverted interrupted suture technique described previously (15), but involves several changes. In detail, the incised dermis was grabbed and folded 10 mm from the incision site using an adson forceps. A 7-0 silk suture needle was inserted perpendicular to the direction of the incision, penetrating the folded dermis. Then, the needle was inserted in the same folded and grabbed manner 10 mm from the incision site on the contralateral side, perpendicular to the incision. When suturing, both sides of epidermis must be in contact in order to prevent reconnection of the superficial lymphatic vessels and capillaries on the fascia. Then, the suture was tied gently twice, minimizing unnecessary tension across the dermis to avoid overly constricting both ends. This folding suture was repeated at 5-mm intervals along the circumferential incision site to minimize exposure of the subcutaneous tissue region, thereby preventing uncontrolled infection (Figure 2).

The immobilization technique, a conventional method to limit the range of motion, was applied to the hindlimb to discourage lymph flow by inhibiting the muscle pump effect of the surrounding muscles. Previous studies (16, 17) have reported that paced contraction of the muscle produces a hydrostatic pressure gradient called a muscle pump effect, which stimulates lymphatic vessel and capillary flow and results in ameliorating lymphedema severity. In this study, instead of using conventional cast immobilization, the calcaneal bursa was attached to the dermis of the upper hip using 7-0 silk in order to create minimal muscle pump effect. Therefore, the induced lymphedema was highly correlated physiologically to lymphedema in humans.

### 2.3 Hindlimb paw thickness measurements

Digital images were obtained biweekly using a high-definition Canon D550 digital camera (Tokyo, Japan). The camera was mounted on a tripod and a precision stainless ruler was placed adjacent to the hindlimb of each mouse for accurate scaling of each obtained image. Hindlimb paw thickness was measured from standardized locations (2 mm from the end of the calcaneus) as described previously (1, 18, 19) using the ImageJ imaging software (US National Institutes of Health, Bethesda).

### 2.4 Lymphography

Lymphography is a fluorescence imaging technique that uses a fluorescence tracer with a specific wavelength as a reagent, paired with a specific band-path filter-mounted camera to visualize lymph flow. In this study, 20 kDa FITC-Dextran was used. All mice were anesthetized using 2% isoflurane inhalation and positioned on the stage of a fluorescence *in vivo* bio-imaging instrument (FOBI; Neoscience, Chungchongbuk-do, Korea). Then, 1% FITC-Dextran in PBS was intradermally injected into the center of the footpad of the both lower limbs. To stimulate lymphatic flow after the injection, we used a cotton swab to apply even pressure on the injection site five times each. Lymphography images were obtained 25 min after injection.

### 2.5 ICG lymphangiography for real-time lymph flow analysis

A high-resolution sCMOS camera (Andor Zyla 4.2 Plus; Oxford Instruments, Abingdon, United Kingdom)—equipped with a band-path filter selectively emitting a range from 810 to 855 nm—combined with a laser-powered light source (M780L2-C4; Thorlabs, New Jersey, USA)—transmitting a range from 750 to 789 nm—was used for real-time lymph flow analysis. Serial image acquisition (exposure time: 0.67 s, number of kinetic series: 100) was performed to enable lymph flow analysis in both lymphatic collecting vessels and capillaries. Signal intensity changes of the collecting lymphatic vessels were collected for quantification of the lymph flow. All mice were anesthetized using 2% isoflurane inhalation and fixed on the stage for evaluation.

Then, an intradermal injection of 10 μL 0.5% ICG in PBS was administered into the center of the footpad using a calibrated infusion pump at a speed of 5 μL/min. Live-time serial images were acquired over a total duration of 20 min starting from the time of the injection. To visually analyze lymphedema status and severity, the Leg Dermal Backflow (LDB) Stage evaluation system, widely used in the assessment of lymphedema patients, was utilized.

After the serial images of ICG lymphangiography images were collected, we selected two representative region of interest (region A and B) of each images as a standard and quantified the intensity changes of entire images from region A to region B [distance(mm)] over number of frames [time(s)] to calculate the speed of lymph flow.

$$\frac{\mathrm{Intensity}\;\mathrm{changes}\;\mathrm{from}\;\mathrm{Point}\;A\;\mathrm{to}\;\mathrm{Point}\;B\;}{\mathrm{Number}\;\mathrm{of}\;\mathrm{frames}}=\frac{\mathrm{Distance}(\mathrm{mm})}{\mathrm{Time}(s)}$$

In this study, time is plotted along the *x*-axis and relative signal intensity along the *y*-axis, as presented in the graphical dataset in the results section.

### 2.6 Tissue sample preparation and histological analysis

After the procedure and follow-up period, histological cross-sectional samples were harvested and prepared from standardized locations as described previously (1, 18, 19) for both hematoxylin and eosin (H&E) staining and immunofluorescence (IF) staining. All samples were prepared as described previously (20). In brief, samples were fixed in 4% paraformaldehyde (Sigma-Aldrich, St. Louis, USA) for 48 h, decalcified in 0.5 M sodium ethylenediaminetetraacetic acid (Sigma-Aldrich, St. Louis, USA) for 48 h, and embedded in paraffin for slide sample preparation.

For histological analysis, all sectioned slide samples were scanned using a Mirax slide scanner (Carl Zeiss, Munich, Germany) and analyzed under brightfield for H&E staining. The images were observed and analyzed using Pannoramic Viewer (3DHistech, Budapest, Hungary). Dermal and subcutaneous thickness was measured at the points of greatest thickness in each cross-section. The number of collecting lymphatics was quantified by counting the vessels in standardized areas (1, 18, 19), with the same scale per high-power field (HPF), measuring 0.25 mm^2^ and analyzing four sections per animal in each group.

For immunofluorescence staining for confocal microscopic images, paraffin-embedded tissue slides were rehydrated and antigen retrieval was performed. Samples were blocked with 20% goat serum (Sigma-Aldrich, St. Louis, USA) and 80% phosphate-buffered saline (PBS; Thermo-Fischer Scientific, Waltham, USA) for 1 h at room temperature. Afterward, samples were incubated with the indicated primary antibodies at 4 °C overnight, followed by incubating with secondary antibodies at room temperature for 2 h. The following primary antibodies were used for the cell staining: anti-LYVE-1 (rabbit polyclonal, AngioBio) and anti-CD31 (hamster monoclonal, Millipore); Alexa Fluor 488-conjugated and Alexa Fluor 594-conjugated were used as the secondary antibodies. Nuclei were stained with DAPI (Invitrogen) and slides were mounted and imaged using a laser-scanning confocal microscope (LSM880, Carl Zeiss, Munich, Germany).

### 2.7 Statistical analysis

Statistical analysis was performed using SPSS, Version 22.0 (SPSS Inc., Chicago, IL). The diameter of the hindlimb, dermal thickness, and subcutaneous thickness were compared using the paired *t*-test. The mean number of lymphatic vessels per high-power field was compared using Student's *t*-test. All *p*-values were two-sided with statistical significance evaluated at the 0.05 alpha level, and 95% confidence intervals were constructed to assess the precision of the obtained estimates.

## 3 Results

### 3.1 Hindlimb paw thickness measurement

**Figure 3:**
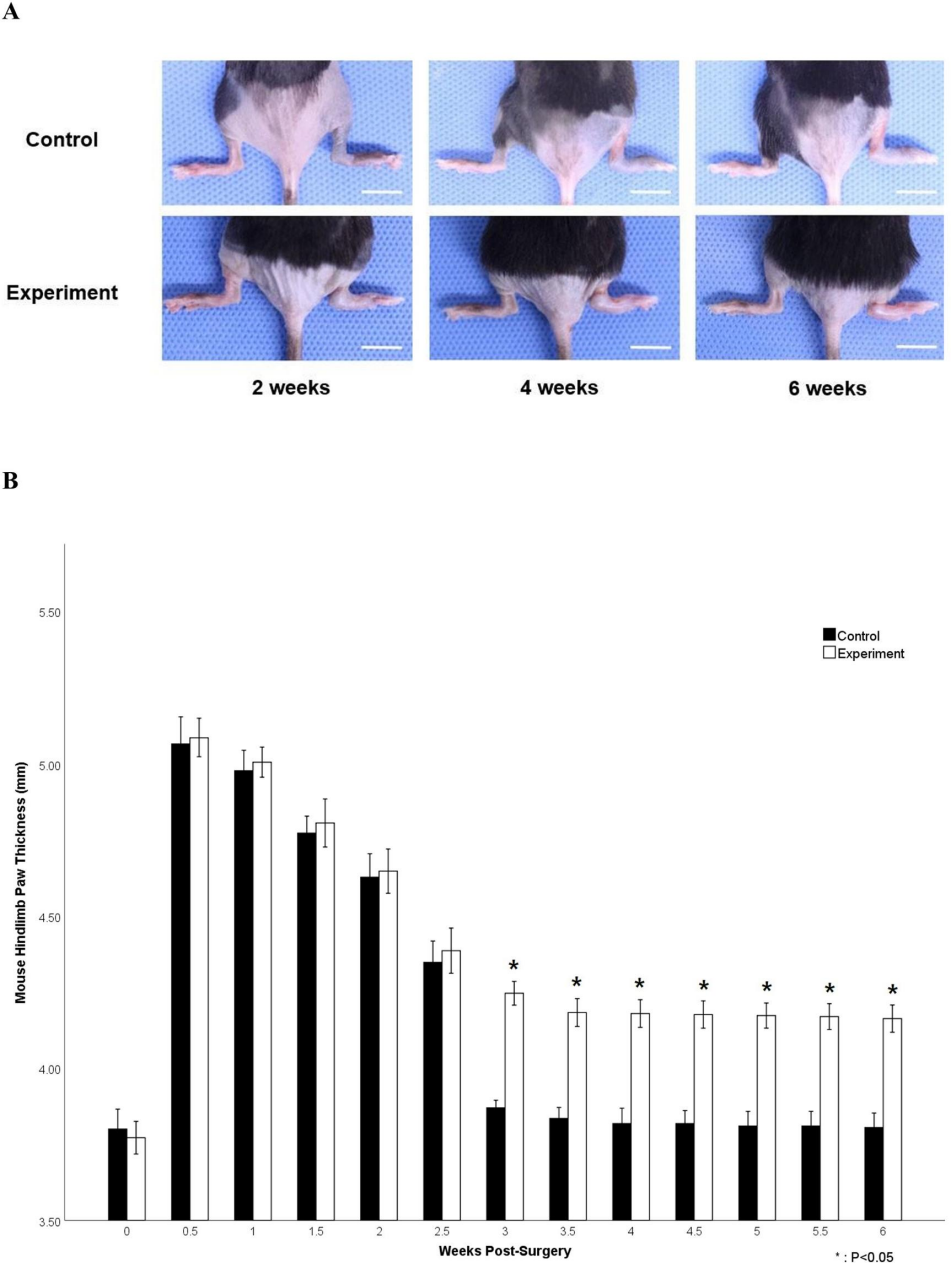
Measurement of hindlimb paw thickness. **(A)** Representative images of changes in mouse hindlimb paw thickness of the control and the experimental groups. Note that 0 week represents before the procedure (scale bar = 10 mm). **(B)** Changes in hindlimb paw diameter after the procedure. Note the significant differences from 3 to 6 weeks after the procedure (*P* < 0.05).

Hindlimb paw thickness increased rapidly immediately after the procedure in both groups and gradually decreased over the following 2.5 weeks, though values were significantly thicker than before the procedure. The rate of decrease varied between groups at each week but was not statistically significant up to 2.5 weeks (5.07 ± 0.08 mm vs. 5.09 ± 0.06 at 0.5 weeks; 4.98 ± 0.06 vs. 5.01 ± 0.05 at 1 week; 4.77 ± 0.05 vs. 4.81 ± 0.08 at 1.5 weeks; 4.63 ± 0.07 vs. 4.65 ± 0.07 at 2 weeks; 4.35 ± 0.07 vs. 4.39 ± 0.07 at 2.5 weeks). However, paw thickness in the control group rapidly decreased at 3 weeks and became similar to the thickness before the procedure (3.80 ± 0.06 at 0 week; 3.87 ± 0.02 at 3 weeks). In contrast, paw thickness in the experimental group also gradually decreased up to 3.5 weeks, but the decrease was significant from 3 to 6 weeks compared to the control group (3.87 ± 0.02 mm vs. 4.25 ± 0.04 at 3 weeks; 3.84 ± 0.03 vs. 4.18 ± 0.04 at 3.5 weeks; 3.81 ± 0.04 vs. 4.18 ± 0.04 at 4 weeks; 3.82 ± 0.04 vs. 4.17 ± 0.04 at 4.5 weeks; 3.81 ± 0.05 vs. 4.17 ± 0.04 at 5 weeks; 3.81 ± 0.05 vs. 4.17 ± 0.04 at 5.5 weeks; 3.81 ± 0.05 vs. 4.16 ± 0.04 at 6 weeks, *P* < 0.05) (Figure 3).

### 3.2 Dermal layer thickness in hindlimb paw

**Figure 4:**
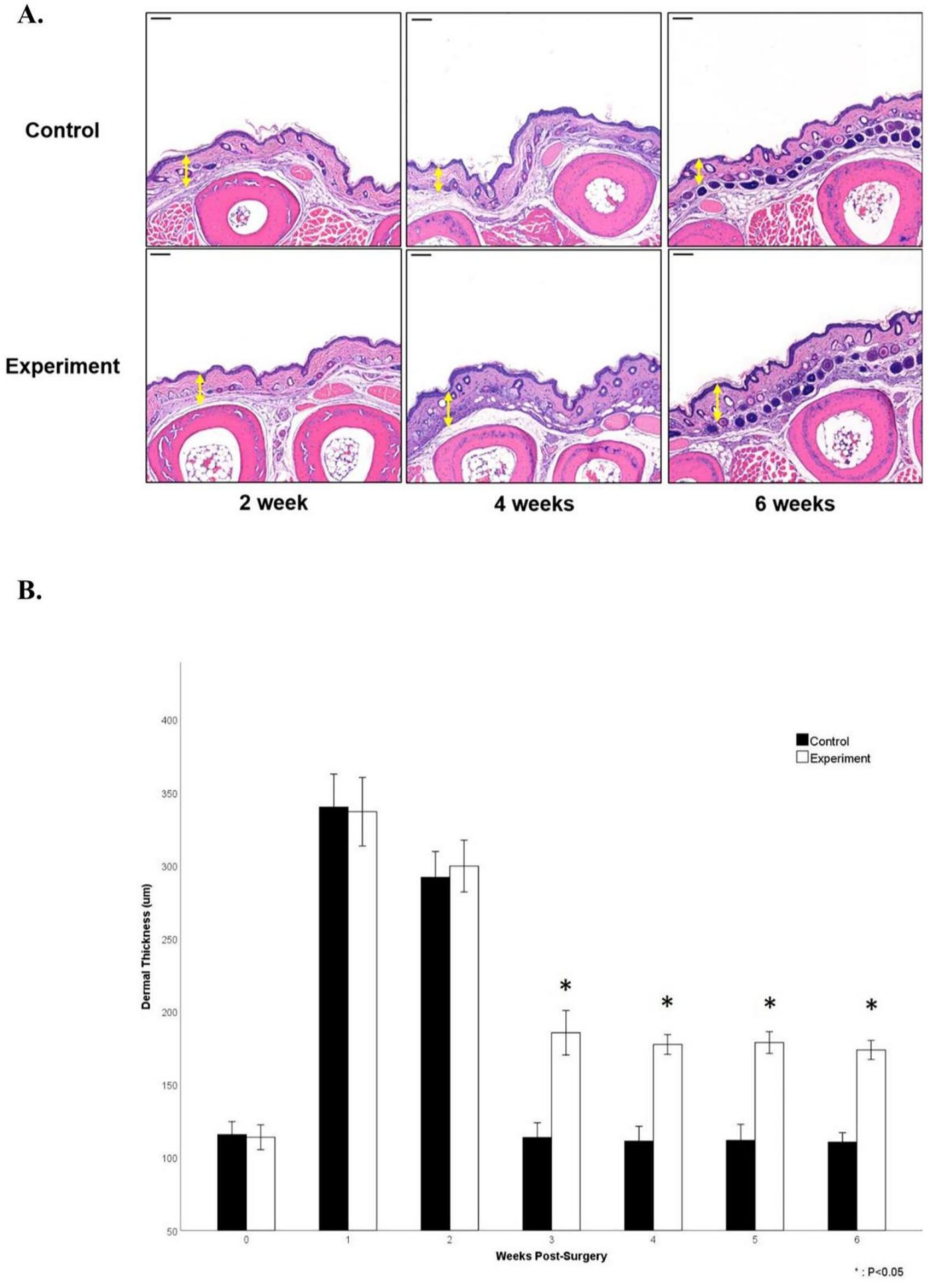
Evaluation of dermal and subcutaneous layer thickness in hindlimb paw. **(A)** Representative H&E staining histological images of mouse hindlimb paw of the control and the experimental groups (scale bar = 100 μm). **(B)** Changes in dermal layer thickness of the hindlimb paw after the procedure. Note the significant differences from 3 to 6 weeks after the procedure.

Dermal layer thickness in both groups increased rapidly after the procedure, followed by a slight decrease at 1 and 2 weeks. Then, thickness in both groups dramatically decreased at 3 weeks and thickness of the control group became statistically not significant before the procedure (115.6 ± 8.5 at 0 week; 113.6 ± 9.6 at 3 weeks). However, the thickness of the experimental group was statistically significant from 3 to 6 weeks (113.6 ± 9.6 vs. 185.4 ± 14.5 at 3 weeks; 111.1 ± 9.7 vs. 177.3 ± 6.5 at 4 weeks; 111.6 vs. 10.5 vs. 178.6 ± 7.1 at 5 weeks; 110.4 ± 6.2 vs. 173.6 ± 6.2 at 6 weeks, *P* < 0.05) (Figures 4A,B).

### 3.3 Lymphography

**Figure 5:**
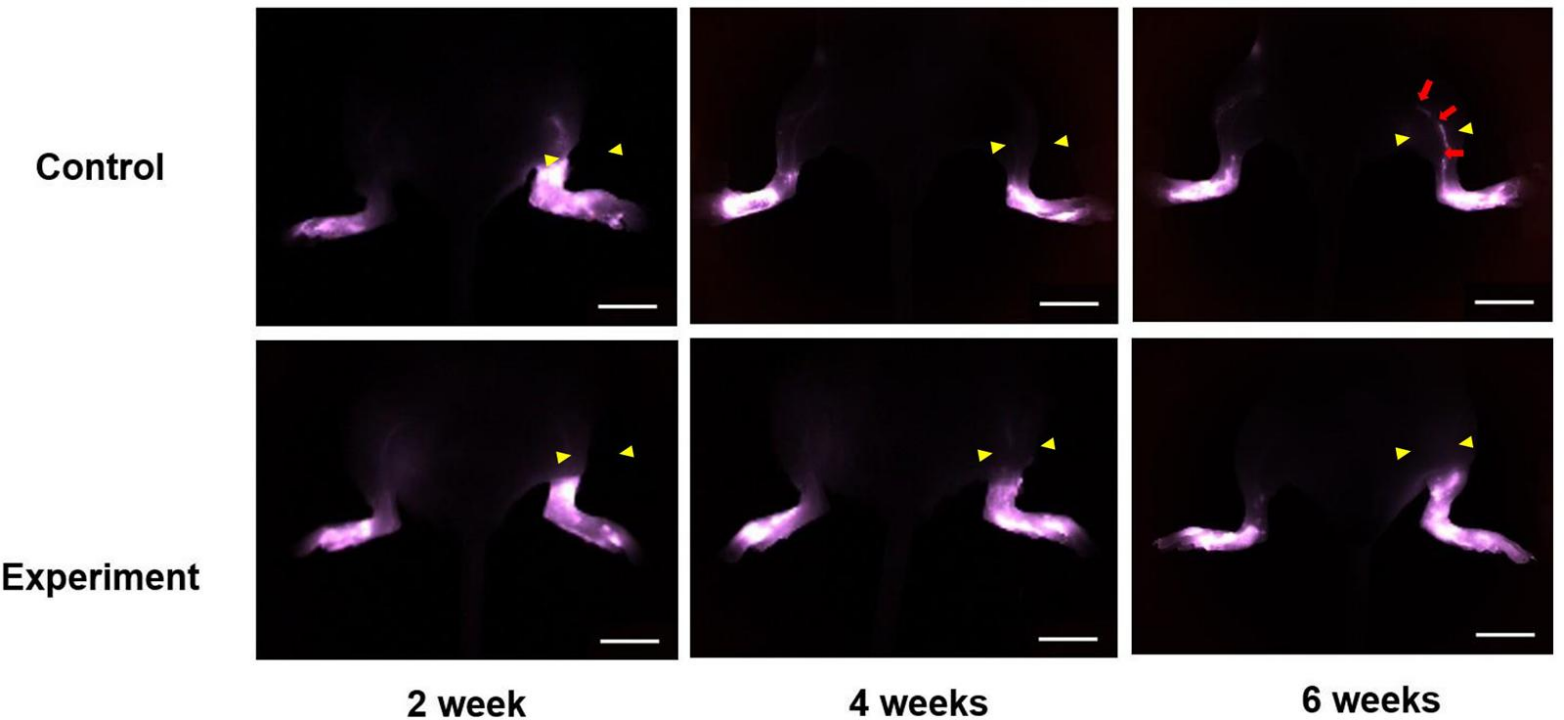
Lymphography was performed to observe lymph flow and lymphatic drainage problems. Both groups showed lymphatic drainage obstruction distal to the surgery site (arrow head) at 2 weeks after the procedure but the control group showed ameliorated lymph flow from 4 weeks, and regenerated deep collecting lymphatic vessel (arrows) was observed at 6 weeks. In contrast, the experimental group showed consistent lymphatic drainage obstruction at 2, 4, and 6 weeks (scale bar = 10 mm).

The experimental group exhibited markedly greater lymphatic drainage congestion of FITC-Dextran tracers distal to the surgery site at 2, 4, and 6 weeks after the procedure. In contrast, the control group demonstrated significant lymphatic drainage obstruction at 2 weeks after the procedure, but this obstruction was resolved. At 2 and 4 weeks, tracer accumulation was restricted to the injection site, and by 6 weeks, regenerated deep collecting lymphatic vessels were observed (Figure 5). Notably, when the control group recovered after 2 weeks, high-intensity signals were observed only at the injection site, whereas in the experimental group, high-intensity signals were observed throughout the distal hindlimb region at the incision site.

### 3.4 ICG lymphangiography

**Figure 6:**
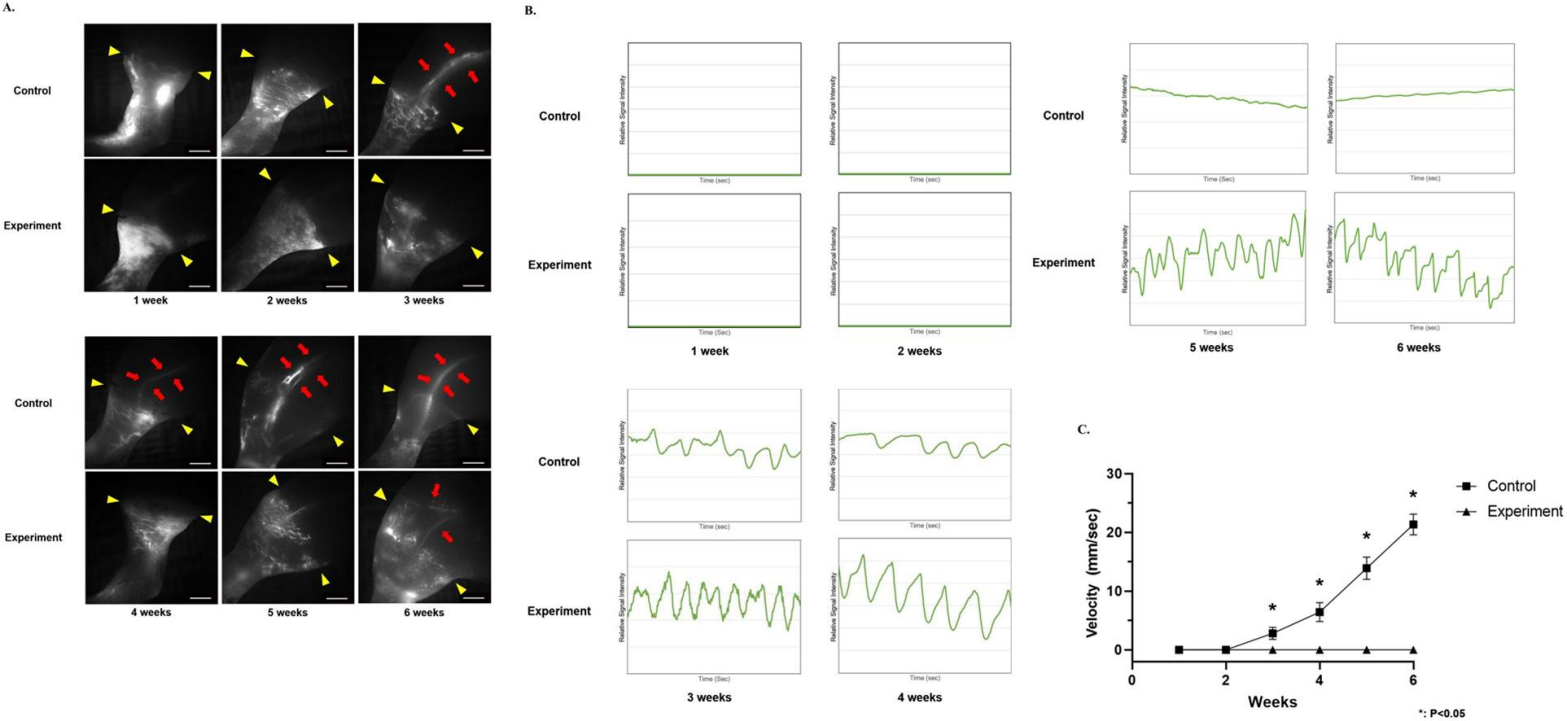
ICG lymphangiography. **(A)** Representative images for real-time lymph flow analysis and high-resolution visual mapping of lymphatic system. Surgery sites are marked as yellow arrows head and regenerated lymphatic vessels are marked as red arrows (scale bar = 2 mm). **(B)** Evaluation of severity of dermal backflow, using signal intensity changes of the superficial lymphatic vessels in the hindlimb, was performed after the procedure. Note that quantification of dermal backflow was made using signal intensity changes (relative signal intensity) over time (per second). **(C)** Measurement of lymph flow velocity in the major collecting lymphatic vessel in the hindlimb after the procedure. Note that velocity represents travel speed of lymph flow with vector value, direction of lymph flow is from distal to proximal side of the hindlimb.

According to ICG lymphangiography (Figure 6A), both groups exhibited lymphatic drainage obstruction distal to the surgery site (yellow arrow head) with no major lymphatic vessel observed at an early stage (at 1 and 2 weeks). Complete saturation of the dermis was observed and no dermal backflow signal was detected at 1 and 2 weeks after the procedure. However, the control group showed ameliorated progression of lymphedema and regeneration of the lymphatic vessels (red arrow) in the region proximal to the surgery site (yellow arrow head) at 3 weeks. Significant amelioration of lymphedema was observed, and dermal backflow was significantly decreased from 4 to 6 weeks. In contrast, the experimental group showed severe lymphedema and obstruction of lymph flow distal to the surgery site (red arrow) up to 5 weeks. Minor regeneration of the lymphatic vessels was observed at 6 weeks, but the size of the lymphatic vessels and their signal intensity were not significant. Moreover, significant dermal backflow was observed from 1 to 6 weeks.

Evaluation of the signal intensity changes in the superficial lymphatic vessels was performed to validate lymphedema severity (Figure 6B). At 1 and 2 weeks, both groups showed complete diffusion (fully saturated signal intensity) of the lymph flow in the superficial lymphatic system with no signal intensity changes. At 3 weeks, the fully diffused lymph flow was ameliorated and dermal backflow was successfully observed in both groups. From 3 to 4 weeks, signal intensity changes in the experimental group were significantly greater than those in the control group (20,166.48–19,051.84, Δ1,114.64 vs. 13,652.52–11,808.01, Δ1,844.51 at 3 weeks; 9,877.62–9,040.60, Δ837.02 vs. 17,303.67–15,525.00, Δ1,778.67). From 5 to 6 weeks, signal intensity changes in the control group showed a 91.01% decrease of the dermal backflow from 3 to 6 weeks. On the other hand, the experimental group showed significant signal intensity changes of the dermal backflow up to 6 weeks (10,170.20–9,946.03, Δ224.17 vs. 45,067.75–44,043.35, Δ1,024.40 at 5 weeks; 1,670.32–1,770.55, Δ100.23 vs. 16,325.69–14,225.46, Δ2,100.22 at 6 weeks).

The velocity of the lymph flow in the major collecting lymphatic vessel in the hindlimb was measured after the procedure to evaluate successful reconnection of the lymphatic system and its transport capacity (Figure 6C). Neither group demonstrated lymph flow in the collecting lymphatic vessel up to 2 weeks. From 3 weeks, lymph flow was observed in the control group, which gradually increased in velocity up to 6 weeks (2.81 ± 1.03 mm/s at 3 weeks; 6.43 ± 1.61 mm/s at 4 weeks; 13.90 ± 1.89 mm/s at 5 weeks; 21.35 ± 1.76 mm/s at 6 weeks). However, no lymph flow was observed in the experimental group, and velocity of the lymph flow was 0 mm/s from 1 to 6 weeks.

For an in-depth, thorough evaluation of lymphedema severity and its physiological condition, LDB Stage evaluation—one of the most widely used methods for assessment of clinical lymphedema patients—was performed (Tables 1 and 2). Both groups demonstrated a significantly high degree of LDB Stage up to 2 weeks (5 vs. 5 at 1 week; 4 vs. 5 at 2 weeks), with slightly more severe lymphedema observed in the experimental group. From 3 weeks onward, the control group demonstrated significant amelioration of lymphedema, reaching Stage 1 by 5 and 6 weeks. In contrast, the experimental group maintained a significantly higher LDB Stage at 3 weeks, with slight amelioration observed from 4 weeks onward. Lymphedema severity persisted up to 6 weeks, with a significant stardust pattern (2 vs. 5 at 3 weeks; 2 vs. 4 at 4 weeks; 1 vs. 4 at 5 weeks; 1 vs. 3 at 6 weeks). Note that details of each LDB stage are description was made in Table 2.

**Table 1:** LDB stage evaluation for progression of lymphedema.

	1 week	2 weeks	3 weeks	4 weeks	5 weeks	6 weeks
Control	5	4	2	2	1	1
Experiment	5	5	5	4	4	3

**Table 2:** LDB stage.

Lymphedema stage	Descriptions
Stage 0	Linear pattern only
Stage I	Linear pattern + splash pattern (around the groin)
Stage II	Linear + stardust pattern (one region)
Stage III	Linear pattern + stardust pattern (two regions)
Stage IV	Linear pattern + stardust pattern (three regions)
Stage V	Stardust pattern (associated with diffuse pattern)

### 3.5 Immunofluorescence confocal imaging

**Figure 7:**
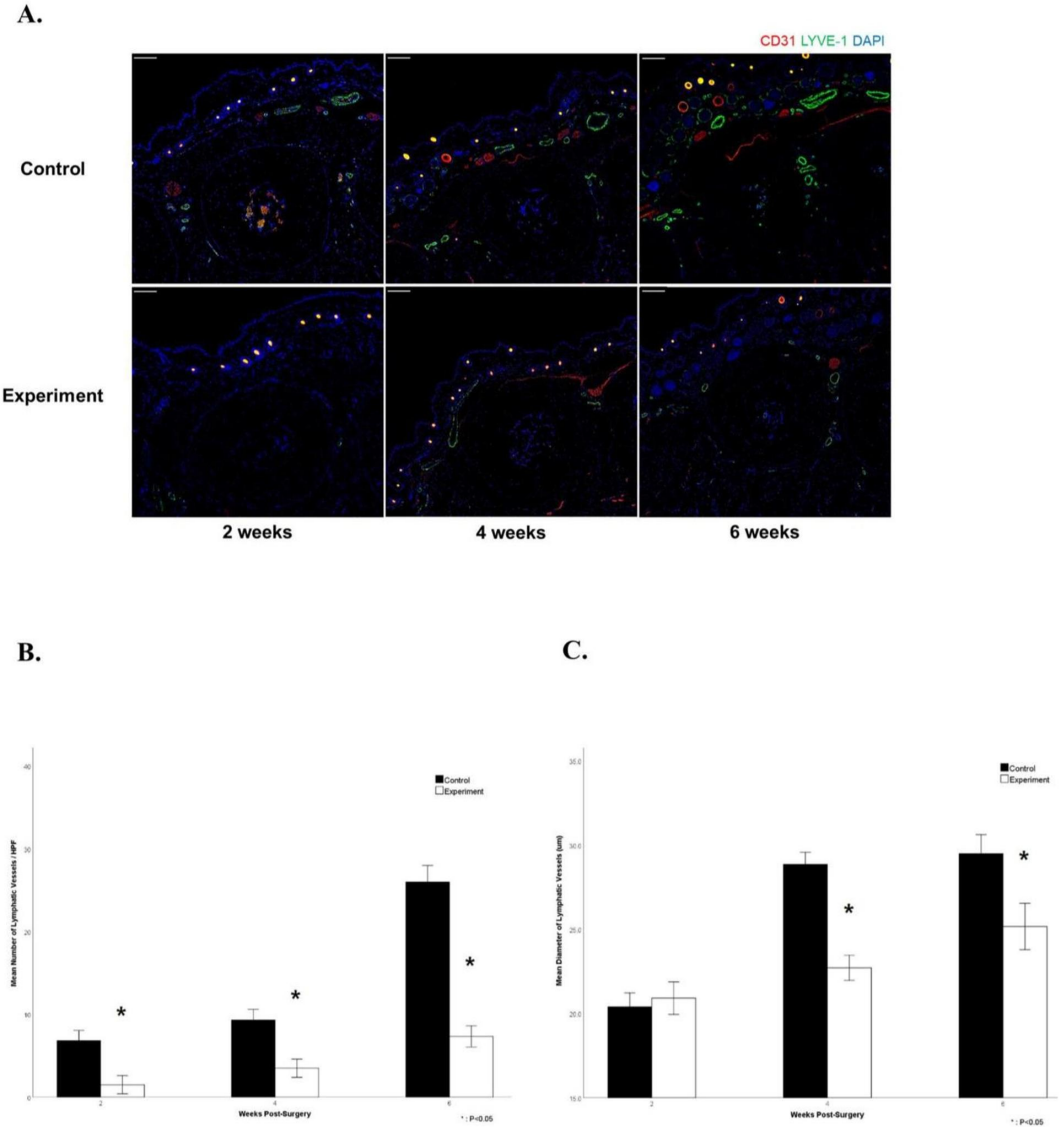
Immunofluorescence confocal images of the hindlimb paw **(A)** confocal microscopic images of the hindlimb paw and comparisons of expressions in CD31 (red), LYVE (green), and DAPI (blue) at 2, 4, and 6 weeks after the procedure (scale bar = 100 μm). **(B)** Mean number of regenerated lymphatic vessels per HPF at 2, 4, and 6 weeks. Note the significantly higher mean number of regenerated lymphatic vessels in the treatment group than the control group. (**C**) Mean diameter of lymphatic vessels at 2, 4, and 6 weeks. Note the significantly greater mean diameter of lymphatic vessels in the treatment group than the control group.

The experimental group demonstrated a significantly lower mean number of lymphatic vessels (green) per HPF, reduced by 6-fold, 3-fold, and 3.7-fold at 2, 4, and 6 weeks after the procedure, respectively (6.83 ± 1.17 vs. 1.50 ± 1.05 at 2 weeks; 9.33 ± 1.21 vs. 3.50 ± 1.05 at 4 weeks; 26.00 ± 1.90 vs. 7.33 ± 1.21 at 6 weeks, *P* < 0.05). In contrast, the control group exhibited a significant increase in both mean number and mean luminal diameter of the lymphatic vessels (Figure 7). This increase in number of collecting vessels and mean luminal diameter corresponded with a decrease in dermal and subcutaneous layer thickness.

For mean lymphatic vessel diameter, the experimental group exhibited statistically significant results from 2 to 6 weeks (20.41 ± 0.78 vs. 20.92 ± 0.92 at 2 weeks; 28.86 ± 0.68 vs. 22.72 ± 0.71 at 4 weeks; 29.49 ± 1.08 vs. 25.18 ± 1.31 at 6 weeks, *P* < 0.05). Regenerated lymphatic vessels in the control group had round, uniform shapes. However, regenerated lymphatic vessels in the experimental group had distorted and tortuous shapes.

## 4 Discussion

In this study, we successfully established a long-term secondary lymphedema hindlimb model in non-transgenic wild-type mice by combining a newly developed surgical technique, controlled radiation exposure, and an immobilization technique. Animal models from previous studies (1, 2, 6, 7, 12, 18) primarily focused on disrupting collecting lymphatic vessels to create secondary lymphedema; however, a major limitation was that the severity and duration of lymphedema did not fulfil the requirements of a standardized lymphedema animal model. The newly developed folding suture technique was effective in blocking lymph flow in the superficial lymphatic system in an early stage, successfully facilitating both duration and severity of lymphedema. Moreover, controlled radiation exposure was performed to inhibit lymphangiogenesis in an early stage to induce and maintain the severity of lymphedema. The immobilization technique significantly reduced lymphatic pumping capability and encouraged its severity, causing irreversible, severe, and chronic lymphedema in the mouse hindlimb. Although we initially proposed to observe chronic lymphedema over 8 weeks, the IACUC restricted the study duration to 8 weeks due to animal welfare considerations. Further studies with significantly longer durations are required to validate long-term chronic lymphedema status.

The significance of this study lies in the successful induction of lymphedema through complete blockage of lymph flow at an early stage. All procedures employed in this study (surgical methods, controlled radiation, and immobilization technique) were inspired and motivated by clinical observation and lymphedema patient experiences. Surgical removal of the lymphatic structure was modeled after cancer resection, which is a prominent cause of lymphedema (4, 5). Controlled radiation was derived from radiation therapy, one of the most common therapeutic procedures for most cancer patients, which may inhibit lymphangiogenesis and radiation fibrosis. Immobilization was improvised from one of the most common behaviors observed in severe lymphedema patients after cancer surgery. The novelty of this study is that all procedures and methods were genuinely inspired by clinical lymphedema patients and their environmental backgrounds.

Another significant aspect of this study was that both duration and severity of the secondary lymphedema significantly improved compared to previously established animal models. Establishing a lymphedema preclinical model has been a significant challenge for decades. Numerous previous studies attempted various methods to create severe and persistent lymphedema. Various techniques and strategies were designed to create lymphedema; however, a major limitation of previous studies (1, 2, 6, 7, 12, 18) was that lymphedema lasted up to 2 weeks only, as it was likely to be induced by a surgical trauma rather than a lymphatic drainage problem and/or disruption of the lymphatic system. By definition, secondary lymphedema is a prolonged drainage problem of the lymphatic system caused by accumulation of lymph flow and unsuccessfully drained lymph fluids. This causes further problems, such as fibrotic tissue changes and cascading damaging effects on edematous region and its surroundings, resulting in irreversible changes to the lymphatic system and surrounding tissue. To overcome limitations in duration and severity of lymphedema, other studies (21, 22) used different strategies in creating lymphedema, such as using a transgenic mouse or genetically knock-out mouse strain to sustain lymphedema over a longer term. These models successfully impaired the lymphatic system and resulted in significant lymphatic drainage problems. However, these models poorly reflect clinical lymphedema patients because the major cause of lymphedema was not the physical disruption of the lymphatic system by surgical procedure. This low correlation of this model with clinical cases of secondary lymphedema patients causes not only disqualified for the standardized secondary lymphedema animal model but also hinders further therapeutic and translational research of secondary lymphedema.

To solidify our results, we used various cutting-edge methods not only to evaluate the severity of lymphedema but also to validate that the lymphedema was induced by the disruption of the lymphatic system and its drainage problem. First, both FITC-Dextran lymphography and high-resolution ICG lymphangiography were performed to thoroughly analyze the status of the lymphatic system during progression of lymphedema. FITC-Dextran lymphography was performed to provide a macro-level view of the entire lower extremities with accumulation of lymph flow so that drainage dysfunction of the lymphatic system was intuitively observed and evaluated. In contrast, high-resolution ICG lymphangiography was performed allowed visual mapping of the lymphatic flow of the collecting lymphatic system and superficial lymphatic system. This facilitated analysis of lymphangiogenesis, reconnection of the lymphatic drainage system, and dermal backflow of the lymph flow before reconnection. As a result, multi-lymphatic drainage system analysis validated that severity of dermal backflow was significantly reduced after the collecting lymphatic system was restored by lymphangiogenesis. Second, we used immunofluorescence confocal microscopic images for both quantitative and qualitative histological systematic analysis of regenerated lymphatic vessels after the procedure. Lymph flow capacity is primarily determined by the number of lymphatic vessels and their condition (1). Based on serial evaluation of regenerated lymphatic vessels at the surgery site, both the number of regenerated lymphatic vessels and their mean diameter were significantly greater in the control group than the experimental group over time. This result indicates that the primary cause of severe edema is disruption of the lymphatic system and unsuccessful lymphangiogenesis. This finding validates that the newly established lymphedema hindlimb model possesses genuine significance and novelty compared to previous ones.

There are some limitations to this study. First, fibrotic tissue changes in the dermis were not evaluated in this study. Previous studies (1, 23) reported that fibrotic tissue changes after the procedure greatly affect progression of secondary lymphedema by inhibiting regeneration of lymphatic vessels and capillaries. Based on IF images of the hindlimb, the regenerated lymphatic vessels in the experimental group were distorted and tortuous compared to those of the control group, resulting in a decrease of the luminal diameter. This may have occurred due to fibrotic tissue changes in the hindlimb, thereby requiring further studies to better understand this phenomenon. Second, further mechanism studies may be needed for in-depth evaluation of the progression of lymphedema. We performed visualization of the lymph flow and regeneration of the lymphatic vessels to demonstrate the role of the newly developed methods in establishing long-term lymphedema. However, cellular-level analyses—including recruitment of DC trafficking and T cell-mediated immune responses—may be required for pathophysiological evaluation of the study. Prior studies (7, 17, 20) have reported that T cells play an important role in the development of chronic tissue inflammation in lymphedema. Therefore, further mechanism studies may be required to clarify progression of lymphedema in the future. Third, further studies may be required to evaluate the efficacy of immobilization. Unlike the cardiovascular system, the lymphatic system is absent in muscles and cannot create contractility and pumping action (24, 25). Instead, the surrounding muscle fibers facilitate lymph flow, so muscle activity significantly influences total lymphatic transport capacity (1, 26, 27). In this study, we performed immobilization of the mice in order to limit mechanical stimulation in pumping of the lymphatic system. Based on the results, immobilization successfully facilitated lymphedema in both duration and severity. However, the extent to which muscle movement was decreased due to immobilization was not evaluated or quantified. Future studies should evaluate and analyze the effects of immobilization on the progression and development of lymphedema in order to expedite understanding of the pathophysiology of secondary lymphedema.

## 5 Conclusion

This study successfully established a long-term secondary lymphedema animal model in a rodent hindlimb. This model was created using a combination of a novel surgical technique, controlled radiation exposure, and an immobilization technique. The significance of this study lies in the fact that it was not only successful in creating lymphedema over a longer term but also created methodological procedures that share high similarities to human lymphedema patients. The novel suture technique in a surgical process that caused significant disruption of both the superficial lymphatic system and the deep lymphatic system and successfully mimicked naturally occurring lymphatic drainage problems, significantly delaying recovery of the lymph flow. Controlled radiation exposure inhibited lymphangiogenesis in the early stage of lymphedema and resulted in irreversible, severe, and chronic lymphedema. After the procedure, the immobilization technique significantly decreased the lymphatic pumping capability, inducing and maintaining the severity of lymphedema. Based on various evaluation methods including visual assessment, immunofluorescence images, lymphography, and ICG real-time lymphangiography, edema was determined to be solely caused by disruption of the lymphatic system and impaired lymph flow transport capacity due to the significant decrease of lymphangiogenesis. This validates that the edema observed in the experimental group is indeed secondary, chronic lymphedema. For further evaluation of lymphedema severity to solidify our results, LDB Stage evaluation—one of the most widely used methods for assessment of clinical lymphedema patients—was performed for additional validation of severity of lymphedema and its physiological condition. This demonstrated the significantly greater severity of lymphedema in the newly established lymphedema hindlimb model.

This newly established lymphedema hindlimb model offers significant improvements in both duration and severity of lymphedema over previously developed animal models. The high correlation with clinical observations supports its potential as a newly standardized animal model for further pathophysiological studies of lymphedema. Moreover, this standardized animal model enables in-depth translational and preclinical research, offering a foundation to develop therapeutic strategies for clinical patients.

## Data Availability

The datasets presented in this study can be found in online repositories. The names of the repository/repositories and accession number(s) can be found in the article/Supplementary Material.
